# The factors contributing to the total radiation exposure of patients during uterine artery embolisation

**DOI:** 10.1002/jmrs.347

**Published:** 2019-07-22

**Authors:** Don J. Nocum, John Robinson, Eisen Liang, Nadine Thompson, Warren Reed

**Affiliations:** ^1^ San Radiology & Nuclear Medicine Sydney Adventist Hospital Wahroonga New South Wales Australia; ^2^ Faculty of Medicine and Health Adventist Hospital Clinical School University of Sydney Wahroonga New South Wales Australia; ^3^ Faculty of Health Sciences, School of Medical Radiation Sciences University of Sydney Cumberland New South Wales Australia

**Keywords:** Angiography, dose, radiation effects, radiology, vascular interventional

## Abstract

Uterine artery embolisation (UAE) is an interventional angiography procedure for the treatment of symptomatic fibroids and/or adenomyosis in women. UAE is a less invasive and non‐surgical alternative to hysterectomy or myomectomy. However, ionising radiation is used for both fluoroscopic and angiographic image guidance to visualise and access the uterine arteries for embolisation and treatment of these benign conditions. Identifying the contributors and implementing dose reduction techniques are particularly important as UAE patients are usually of child‐bearing age. The purpose of this review was to examine the progression of literature on radiation exposure measurements and identifying the factors contributing to the total radiation exposure of female patients undergoing UAE. A Medline, ProQuest Central, ScienceDirect and Scopus database search from 2000 to 2018 was performed and forty articles were deemed acceptable for review following the inclusion and exclusion criteria set. UAE is a viable alternative to hysterectomy and myomectomy, as the reviewed literature demonstrated that the reported radiation exposure doses appear to be below the threshold for any deterministic radiation risks. The total radiation exposure of UAE patients is affected independently by multiple patient, operator expertise and technique, angiographic imaging and x‐ray unit variables. Uterus preservation can be attained post‐UAE with dose reduction and optimisation, however, a longitudinal study on UAE patients and their risk of radiation‐induced deterministic and/or stochastic effects is recommended.

## Introduction

Uterine Artery Embolisation (UAE) is an interventional procedure routinely performed in an angiography suite equipped with an x‐ray image intensifier or flat–panel detector unit. UAE is a form of treatment for women suffering from symptomatic uterine fibroids, also referred to as uterine fibroid embolisation (UFE) or adenomyosis. Uterine fibroids are benign growths in the uterus that can cause menorrhagia and bulk–related symptoms of pelvic pressure and bladder compression.^1^, ^2^, ^3^ Adenomyosis is also a benign disease of the uterus, where UAE can treat heavy menstrual bleeding (HMB) and dysmenorrhoea.^4^

UAE has been independently evaluated in literature for the efficacy and safety in the treatment of symptomatic uterine fibroids and adenomyosis and as a non‐surgical alternative to hysterectomy and laparoscopic myomectomy.^5^, ^6^ Uterine fibroids and adenomyosis are the most common indication for hysterectomy, however, the main disadvantage of this operation is the elimination of the possibility of future pregnancy for the patient.^7^, ^8^ Myomectomy is another surgical option for fibroids that preserves the uterus. This operation is technically more challenging than a hysterectomy and the probability of fibroids recurrence at 5 years post‐myomectomy is approximately fifty percent.^9^ The advantages of UAE over surgical approaches include: a minimally invasive technique, general anaesthetic is not required, is a shorter procedure time (1–2 h), requires a shorter hospital stay (one to two nights), shorter recovery period, reduced infection risk, lower hospital and patient costs, offers the potential of retained fertility and multiple fibroids and/or adenomyosis are treated with a single procedure and uterus preservation.^10^ The main disadvantage is the use of ionising radiation for the imaging guidance during the procedure to visualise the vasculature and angiographic devices for embolisation and treatment of the fibroids and/or adenomyosis. Other potential complications of the UAE procedure are injury to the artery (<1%) and infection (1–3%).^11^

Research on the topic of radiation dose contributors and minimising radiation exposure during UAE is particularly significant as the patients involved are usually of child–bearing age. Their reproductive organs are within the primary beam of the x‐rays during the procedure and thus dose reduction strategies are especially important during the treatment of the fibroids or adenomyosis in order to minimise the risk of any deterministic and/or stochastic effects. Compared to the surgical alternatives, UAE aims to preserve the uterus integrity and dose optimisation can further preserve these radiosensitive reproductive organs from ionising radiation.

The scope of this review will discuss the angiographic technique on absorbed dose and effective dose, fluoroscopic and angiographic imaging modes, radiologist and radiographer expertise, radiologist and operator technique and modern angiography x‐ray units. The purpose of this article was to review the literature on the qualitative and quantitative methods used to measure radiation exposure and to determine the factors contributing to the total radiation exposure of patients during UAE.

## Materials and Methods

A Medline, ProQuest Central, Scopus and ScienceDirect database search from 2000 to 2018 using the keywords, ‘uterine fibroid embolisation/embolization’ (UK/American English spelling), ‘uterine artery embolisation/embolization’ (UK/American English spelling) and ‘radiation dose’ was performed. The following inclusion criteria were adopted: (1) research studies on radiation dose and uterine fibroid embolisation procedures; (2) uterine artery embolisation for uterine fibroids and adenomyosis; (3) radiation dose contributors and dose reduction; (4) research performed on human subjects only; and (5) research from >10–15 years to provide a historical review of research progression in this area. The exclusion criteria included: (1) papers published in a language other than English and not translated to English; (2) prophylactic uterine artery embolisation for placenta previa and placenta accreta; and (3) uterine artery embolisation for post‐partum haemorrhage. The organisational pattern of this review follows a historical progression of the literature and is categorised into the different areas that contribute to the total radiation exposure of UAE patients.

**Figure 1:**
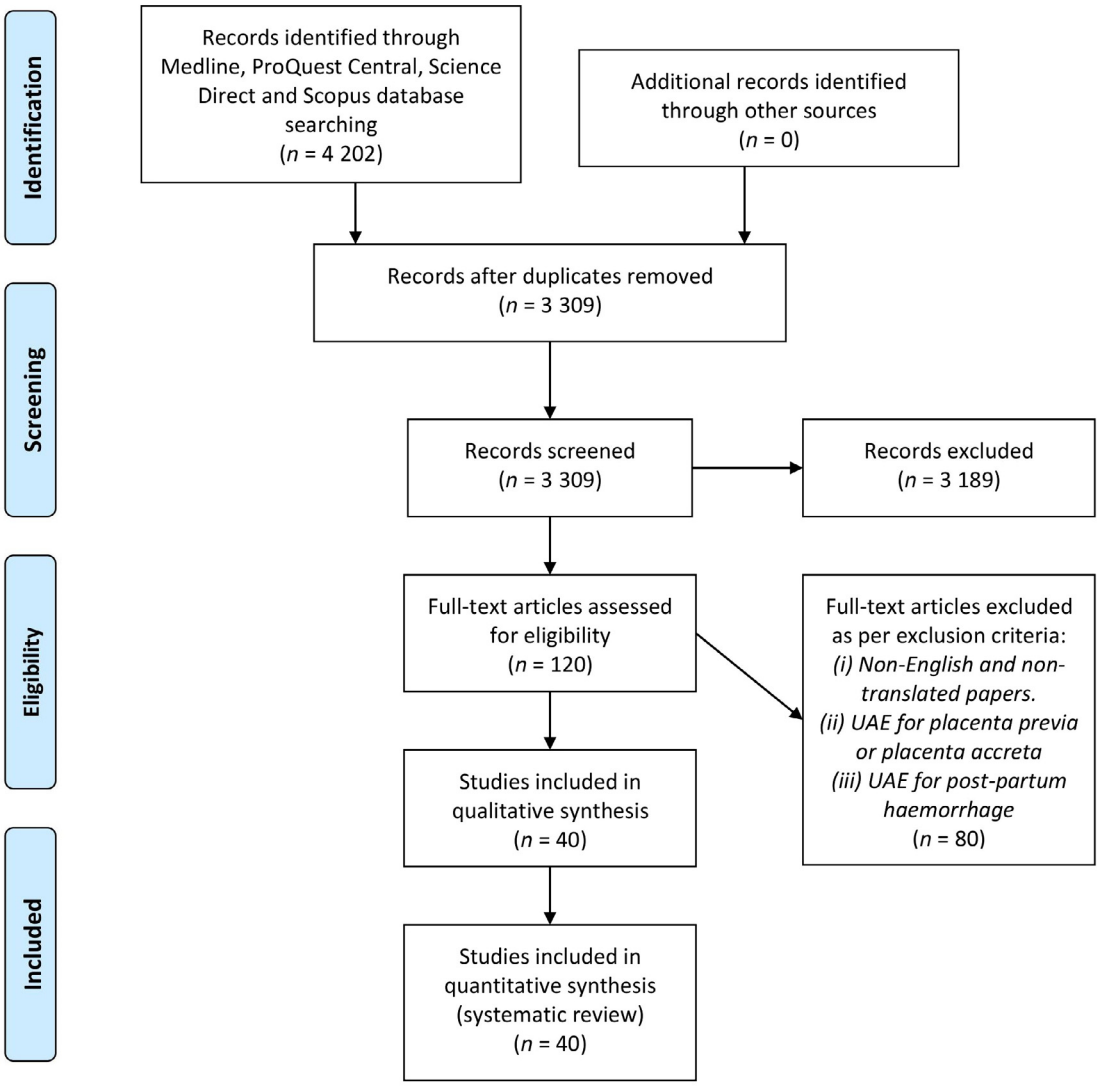
A modified PRISMA flow chart showing the article search, screening and selection process.^12^

A total of 3,309 articles were screened by title, abstract and recency across four databases using the search terms as mentioned in the methodology (Table 1). The results were further refined to specifically include the topic question of patient radiation exposure and thus produced 120 articles from the Medline, ProQuest Central, ScienceDirect and Scopus databases. These four databases were considered adequate due to the expected volume, nature and complexity of the literature to be identified. Following the screening of all titles by most recent, abstracts and full readings, 40 articles were deemed acceptable for review after applying the exclusion criteria. Articles were rejected during the title stage due to their lack of relevance and direct relation to the factors contributing to the total radiation exposure during UAE. Upon analysis of abstracts and full reading, subsequent articles were rejected due to misalignment with the inclusion criteria, the limitations of the literature and the extent to which these limitations affected the study results. Figure 1 shows a modified PRISMA flow chart of the records search and screening process for this literature review.^12^

**Table 1:** Articles produced from the Scopus, ScienceDirect and ProQuest Central databases using the keywords relating to the topic question.

Keywords	Database			
	Medline	ProQuest Central	ScienceDirect	Scopus
‘Uterine fibroid embolisation’ AND ‘radiation dose’	23	428	58	3
‘Uterine fibroid embolization’ AND ‘radiation dose’	24	428	442	32
‘Uterine artery embolisation’ AND ‘radiation dose’	37	936	139	9
‘Uterine artery embolization’ AND ‘radiation dose’	37	428	1103	75

This table demonstrates the search results (in numerical values) using the different keywords and English spelling (UK and American English) from the four databases selected.

## Results

**Figure 2:**
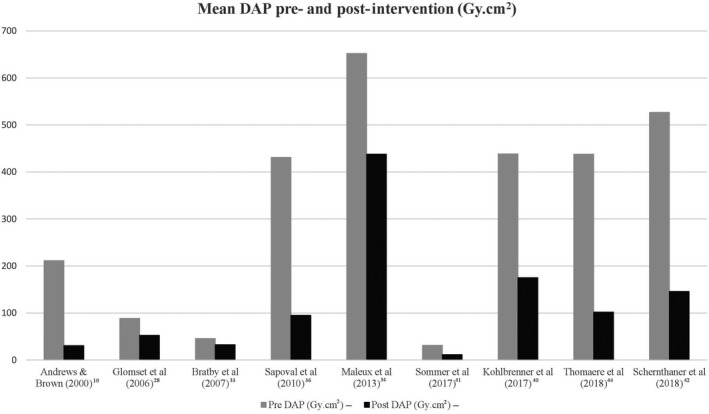
Mean DAP Pre‐ and Post‐Intervention from selected reviewed articles.

**Figure 3:**
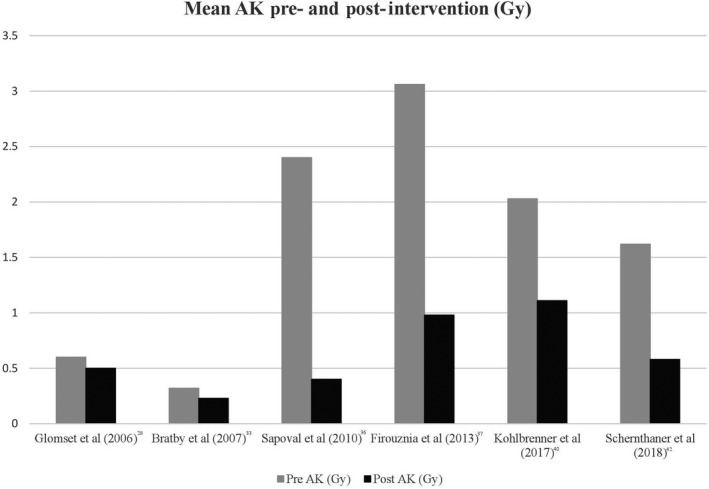
Mean AK Pre‐ and Post‐Intervention from selected reviewed articles.

The review of 40 articles has shown that there are multiple patient, operator, angiographic imaging and x‐ray unit factors that contribute to the total radiation exposure of patients undergoing UAE. Table 2 shows selected articles included in this literature review which provide a brief overview on the radiation dose monitoring methods used during UAE and their respective measured variables and radiation dose exposure values. Table 3 reports the selection of mean DAP (pre‐ and post‐intervention), mean AK (pre‐ and post‐intervention), mean absorbed ovarian dose and mean estimated effective dose from the reviewed literature. Figures 2 and 3 present a graphical representation of the dose reductions for DAP and AK respectively, when an intervention was used. The literature uses DAP (x‐ray unit radiation output), AK (absorbed skin dose) or both to measure patient radiation dose exposure, as shown in these 2 figures.

**Table 2:** Summary of reviewed articles on selected radiation dose measurements, methods and results during UAE.

Author/s	Year	Radiation dose monitoring method/s and measured variables	Selected summary of results
Nikolic *et al* 13	2000	Estimated absorbed radiation doses to the ovaries & skin entrance. Lithium fluoride dosimeter (intracavity and skin surface).	Mean absorbed ovarian dose = 0.22 Gy. Mean absorbed skin dose = 1.62 Gy.
Nikolic *et al* 14	2000	Effects of fluoroscopic imaging techniques (e.g. pulsed fluoroscopy (PF) and non‐pulsed fluoroscopy (NPF)) on Absorbed Ovarian Dose (AOD). Thermoluminescent Dosimeters (TLDs) on anamorphic phantoms.	NPF‐AOD 1.7× higher than PF‐AOD. AOD from oblique magnified fluoroscopy was 1.9× greater. AOD from non‐magnified oblique fluoroscopy was 1.1× greater.
Andrews & Brown^10^	2000	Variables investigated: Fluoroscopy time, number of images acquired, height, weight, dose‐area‐product (DAP) and estimated effective dose (mSv).	Mean DAP decreased from 211.4 to 30.6 Gy cm^2^ using dose reduction techniques. Mean DAP = 4827 Gy cm^2^
Vetter *et al* 17	2004	Absorbed organ and effective doses. Monte Carlo simulation of radiation transport.	Mean DAP = 59.9 Gy cm^2^. Mean absorbed ovarian dose = 0.051 Gy. Mean estimated effective dose = 34 mSv.
Vetter *et al* 27	2005	Mean organ and effective doses and dose conversion coeffecients (DCC) comparing DSA and LIH. Monte Carlo simulation.	Mean DAP = 37.1 Gy cm^2^. Avg DCC for DSA image procedures = 0.572; Men effective dose = 29.6 mSv.
Sapoval *et al* 36	2010	Assessed low–dose and low–frame rate fluoroscopy & angiography on mean peak skin dose, DAP, ovarian and uterus dose and effective dose. Phantom study and TLDs for background measurements and skin–absorbed radiation dose in fixed positions at the beam entrance site.	For low–dose & low–frame rate fluoroscopy: Mean peak skin dose = 0.4 Gy. DAP = 95.15 Gy cm^2^. Dose to ovaries & uterus = 0.083 Gy. Effective dose = 24 mSv.
Firouznia *et al* 37	2013	Ovarian radiation doses comparing flat–panel (FP) and conventional (CV) angiography In‐vitro phantom study calculations and TLDs located on anterior and posterior surface of patient at level of left and right ovaries.	Mean right entrance dose = 1.59 Gy (CV) vs. 0.52 Gy (FP). Mean left entrance dose = 1.47 Gy (CV) vs. 0.46 Gy (FP). Mean right ovarian dose = 0.14 (CV) vs. 0.02 Gy (FP). Mean left ovarian dose = 0.1 (CV) vs. 0.02 Gy (FP).
Das *et al* 32	2015	Variables investigated: mean fluoroscopy time and DAP (UFE performed by trainee radiologists).	Mean fluoroscopy time = 18.4 min (first 5 UAE procedures) vs. 16.3 min (last 5 UAE procedures). Mean DAP = 4827 Gy cm^2^ Mean AK = 0.26 Gy
Kohlbrenner *et al* 40	2017	Compared CKAP and CAK on flat–panel (FP) and conventional (CV) angiography units.	Mean CKAP ↓ 60% = 438.5 Gy cm^2^ (CV) vs. 175.2 Gy cm^2^ (FP). Mean CAK ↓ 45% = 2.03 Gy (CV) vs. 1.11 Gy (FP).
Thomaere *et al* 44	2018	Compared DAP and estimated organ dose on the ovaries and uterus on old imaging platform (Allura Xper) and new imaging platform (AlluraClarity).	Mean total DAP ↓ 77% = 102 Gy cm^2^ (NP) vs. 438 Gy cm^2^ (OP). Mean Ovarian dose ↓ 64% = 0.04 Gy (NP) vs. 0.12 Gy (OP). Mean Uterine dose ↓ 66% = 0.04 Gy (NP) vs. 0.12 Gy (OP).

This table demonstrates a selection of the literature reviewed and the multiple variables that have been investigated regarding the radiation dose exposure of UAE patients. The information and data presented are in order of publication (old to most recent). All measurements are in SI or SI–derived units. PF, pulsed fluoroscopy; NPF, non‐pulsed fluoroscopy; AOD, absorbed ovarian dose; TLD, thermoluminescent dosimeter; DAP, dose‐area‐product; DCC, dose conversion coefficients; DSA, digital subtraction angiography; LIH, last‐image‐hold; FP, flat panel; CV, conventional; UFE, uterine fibroid embolisation; CKAP, cumulative kerma air product; CAK, cumulative air kerma; NP, new platform; OP, old platform.

**Table 3:** Summary of reviewed articles and the calculated DAP and AK (Pre‐ and Post‐Intervention, mean absorbed ovarian dose and mean estimated effective dose.

Article	Pre‐DAP (Gy cm^2^)	Post‐DAP (Gy cm^2^)	Pre‐AK (Gy)	Post‐AK (Gy)	Mean absorbed ovarian dose (Gy)	Mean estimated effective dose (mSv)
Nikolic *et al* 13	–	–	–	1.62	0.22	–
Andrews & Brown^10^	211.4	30.6	–	–	–	–
Vetter *et al* 17	0	59.9	–	–	0.05	34
Vetter *et al* 27	0	37.1	–	–	–	29.6
Glomset *et al* 28	88.6	52.5	0.6	0.5	0.1	22
Bratby *et al* 33	45.77	32.48	0.32	0.23	–	–
Mondshine *et al* 35	–	146.35	–	–	–	–
Sapoval *et al* 36	431.13	95.15	2.4	0.4	0.083	24
Firouznia *et al* 37	–	–	3.06	0.98	–	–
Maleux *et al* 34	652.1	437.9	–	–	0.1187	–
Sommer *et al* 41	31.24	11.59	–	–	–	–
Kohlbrenner *et al* 40	438.5	175.2	2.03	1.11	–	–
Thomaere *et al* 44	438	102	–	–	0.04	–
Schernthaner *et al* 42	526.8	145.9	1.62	0.58	–	–

This table demonstrates the radiation dose results from selected reviewed articles to show the average DAP, AK, mean absorbed ovarian dose and mean estimated effective dose. These calculations justify whether UAE is performed under safe levels of radiation that are below thresholds of radiation–induced injury. The information and data presented are in order of publication (old to most recent). All measurements are in SI or SI–derived units. DAP, dose‐area‐product; AK, air kerma.

## Discussion

### Angiographic technique on absorbed dose and effective dose

In 2000, Nikolic & Spies *et  al* 13 were one of the first authors to publish results on estimated absorbed radiation doses to the ovaries and skin entrance during UAE. Twenty UAE patients were studied and measured by placing lithium fluoride dosimeters, one into the posterior fornix of the vagina and another on the skin at the beam entrance site. It was found that the estimated absorbed ovarian dose for UAE was much greater than for more common fluoroscopic procedures, specifically; hysterosalpingography (0.004–0.006 Gy), fallopian tube recanalisation (0.002–0.028 Gy), and CT of the trunk (0.001–0.019 Gy). At a mean estimated absorbed ovarian dose of 0.223 Gy and a mean absorbed skin dose of 1.623 Gy for UAE, it was deduced that the patient radiation doses were not detrimental as they did not outweigh the risks associated with the examination of pelvic irradiation for Hodgkin disease (2.63–35 Gy).^13^ Nikolic & Abbara *et  al* 14 then evaluated the influence of fluoroscopic imaging techniques, such as pulse fluoroscopy and non‐pulsed fluoroscopy, on the UAE absorbed ovarian dose (AOD). However, TLDs and the measurements of AOD were only performed on an anthropomorphic phantom. The AOD was 1.7 times greater in non‐pulsed fluoroscopy compared to pulsed fluoroscopy (15 and 30 pulses/sec) and the AOD from oblique magnified fluoroscopy and non‐magnified oblique fluoroscopy was 1.9 and 1.1 times greater respectively, compared to non‐magnified posterior‐anterior fluoroscopy.^14^ As the fundamentals of fluoroscopic imaging were being investigated, the concepts of limiting fluoroscopy time, oblique projections and magnification were contributing to the reduction in patient radiation exposure.

Radiation has a linear, no threshold relationship to the dose received by patients undergoing any medical imaging.^15^ The ALARA (‘As Low As Reasonably Achievable’) principle should be utilised during UAE to reduce patient radiation exposure while obtaining acceptable angiographic image quality, without compromising diagnosis and treatment. The UAE absorbed and effective doses should be reduced with low exposure x‐ray imaging and screening times to avoid any possible deterministic effects of ionising radiation. The radiation skin–absorbed dose is recommended to be <2 Gy^16^, as Nikolic & Spies *et  al* 13 demonstrated that their mean AK was below this threshold. Stochastic effects are those that lack a threshold value and the risk of DNA injury increases when more radiation is absorbed by the patient. Stochastic effects are more difficult to determine, but may be indicated by dosimetric quantities such as dose‐area‐product (DAP) and effective dose.^17^ The ICRP Publication 103 defines radiation exposure as the process of being exposed to radiation or radionuclides, where the significance of exposure is determined by the resulting radiation dose.^18^ Hence, the total radiation exposure for UAE patients forms a quantitative measurement of dose but also indicates any risk of radiation–induced effects. UAE predisposes patients to ionising radiation, particularly the patients’ uterus and ovaries which are in direct x‐ray beam for prolonged periods of time. This counteracts the aim of the procedure to preserve these radiosensitive organs and potentially maintain fertility.^19^ It is difficult to determine the likelihood that the dose associated with UAE causes an increased risk related to the patient’s fertility. The known risk of infertility associated with UAE is due to the non‐target embolisation of the ovaries (collateral bed between the ovarian and uterine arteries) or target embolisation of the ovarian artery association with the fibroid.^20^ Multiple variables that are beyond the control of the radiographer and radiologist were identified that contribute to radiation exposure, including; the conversion efficiency of the imaging chain (fluoroscopic equipment), larger body habitus patients and large fibroid mass sizes (incident radiation is proportional to tissue volume and density).^20^ The following radiographer and radiologist controllable variables reduce the overall radiation exposure to the patient; minimising object‐image distance, use of low–dose and/or pulsed fluoroscopy and limiting the use of oblique projections, magnification and digitally subtracted acquisition runs.^19^

Andrews & Brown^10^ investigated the factors responsible for patient radiation exposures and their quantifiable measurements during UAE. Procedure variables including fluoroscopy time, number of images acquired, height, weight, DAP and estimated effective dose (mSv) were measured. The results across 35 patients showed a decrease in mean fluoroscopy time from 30.6 to 14.2 min and mean DAP from 211.4 to 30.6 Gy cm^2^. These values were achieved by employing dose reduction techniques such as avoiding digital subtraction angiography (DSA) acquisitions after the initial aortogram, using last‐image‐hold (LIH) with contrast media injection, aggressive tight collimation and no magnification.^10^ There were some gaps in this study that may have showed some inconsistencies in data collection, such as a relatively small sample size and that the testing performed on four different angiography units that had been tailored to suit operator preferences in fluoroscopy and exposure dose, filtration and frame rates per second. Vetter *et  al* 17 also had a small study cohort of 33 UFE cases but were able to calculate very precise organ doses and effective doses by using the Monte Carlo simulation of radiation transport. Their calculations using the dose conversion factor (DCF) multiplied by the measured DAP showed a mean DAP of 59.9 Gy cm^2^ (median 23.4; range 8.8–317.5 Gy cm^2^), mean absorbed ovarian dose of 51 mGy and a mean estimated effective dose of 34 mSv (median 13; range 5–182 mSv).^17^ From these results, it was found that the average effective dose was approximately double the dose of an abdominal CT examination.^21^ The authors justified that the dose reduction features of angiographic equipment and radiographic techniques as mentioned in previous literature^22^, ^23^, ^24^, ultimately influence the organ dose and effective dose. Scheurig‐Muenkler *et al* 25 had gathered from the UAE and radiation dose research performed inclusively from 2000 to 2013 that the following dose–saving measures were recommended for UAE protocols: optimised source‐object, source‐image and object‐image distances, pulsed fluoroscopy, angiographic runs in posterior‐anterior direction with 0.5 frames per second, no magnification, tight collimation and no additional aortography.^25^, ^26^ This study also concluded that the target DAP for UAE should be maintained below 50 Gy cm^2^.^25^

As the methodology used for radiation exposure monitoring moved from TLD measurements to DAP values, there was no absolute correlation between the two readings for absorbed dose. The DAP is defined as the quantity of radiation delivered over a specified area rather than the amount of radiation absorbed. Hence, the DAP can only estimate specific organ dose if the beam geometry is known and constant.^10^ The technique of placing a TLD in the posterior fornix of the vagina may have been considered accurate for absorbed dose measurements^13^, but was regarded as being invasive and did not provide continuous intra‐procedural feedback on dose.^10^ The literature readily uses DAP values to account for radiation dose attributed by a procedure, as DAP is more functional and can be standardised for patients per procedure.

### Fluoroscopic and angiographic imaging modes

In 2005, Vetter & Schultz *et  al* 27 expanded their study cohort to 70 UAEs and optimised their practice by omitting the use of oblique views and comparing protocols which use LIH versus DSA runs. The Monte Carlo simulation was utilised to calculate the organ and effective doses and dose conversion coefficients (DCC) (mSv Gy cm^2^). For the UAEs with DSA imaging, the mean organ dose for ovaries, uterus and urinary bladder were 0.051, 0.093 and 0.127 Gy respectively. For the UAEs with LIH imaging, the mean organ dose for the ovaries, uterus and urinary bladder was reduced to 0.0078, 0.0091 and 0.0051 Gy respectively.^27^ These findings demonstrated that PA projections and LIH imaging can potentially reduce the radiation dose to the reproductive organs of patients undergoing UAE. A limitation of the study was the uneven distribution of patients that compared DSA (*n *=* *43) and LIH (*n *=* *26) imaging. Glomset *et  al* 28 further researched the radiation exposure to the skin, uterus and ovaries during UAE, but this time compared two different types of angiography systems with different dose levels; one non‐pulsed system with 3.3 mm Al filtering and fixed peak voltage 80 kVp (Advantage; G.E. Medical Systems, Milwaukee, Wisc. USA) and a pulsed system (Angiostat; Siemens AG, Erlangen, Germany) with 5.4 mm Al filtering and fixed peak voltage 80 kVp. The mean DAP for the pulsed system was statistically significant (*P *≤* *0.05), as expected, giving a value of 52.5 Gy cm^2^ compared to a mean DAP for the non‐pulsed system at 88.6 Gy cm^2^.^28^ The ovarian doses were below the threshold for any temporary sterility at 0.6–4 Gy or permanent sterility at 2.5–10 Gy in a single dose and 6 Gy with a protracted exposure.^28^ During this period, these findings were considered unsubstantial in determining any stochastic risk for radiation–induced malignancy and genetic injury to future offspring.

**Figure 4:**
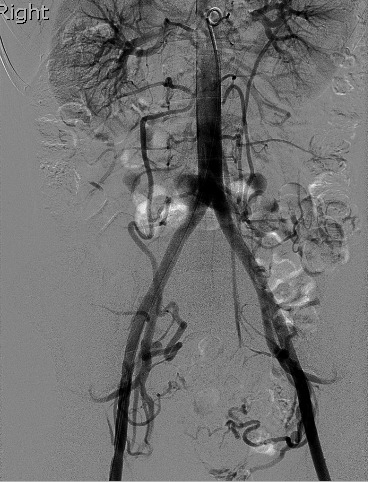
Abdominal aortogram demonstrating bilateral uterine artery supply to the fibroid with a right and left ovarian artery. Source: Property of the Sydney Adventist Hospital, Adventist Healthcare Ltd (2018).

**Figure 5:**
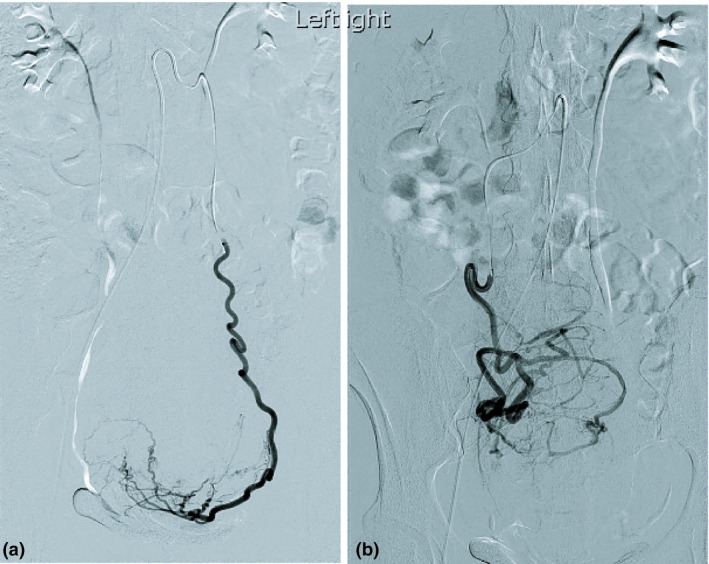
(A) Selective left ovarian artery demonstrating uterine fibroid association. (B) Selective right ovarian artery demonstrating uterine fibroid association. Source: Property of the Sydney Adventist Hospital, Adventist Healthcare Ltd (2018).

White *et  al* 29 published a paper in 2007 which investigated the radiation dose attributed by aortography and DSA acquisition runs. This imaging technique has been routinely used by most radiologists in previous literature.^13^, ^17^, ^20^, ^27^, ^30^ Aortography is a form of digital subtraction angiogram following contrast media injection and its purpose in a UAE procedure is to: (1) demonstrate the roadmap for the uterine arteries supplying the fibroid or adenomyosis and ovarian artery association in the initial angiogram (Figure 4), and (2) assess the collateral arterial supply for supplemental embolisation, post‐uterine artery embolisation on the left (Figure 5a) and on the right (Figure 5b). From the 25 UFE patients involved in this study, one patient had undergone a right‐sided ovarian artery embolisation. It was found that 21% of the total dose to the patient was attributed by the aortography used during the examination.^27^ White* et  al* 29 argued that aortography in UFE requires reconsideration amongst interventional radiologists since less than 6% of patients may benefit from supplemental ovarian artery embolisation and aortography that demonstrates substantial collateral uterine perfusion only benefitted 1% of women who had subsequent collateral embolisation.^29^ White & Banovac *et  al* 30 deduced from their retrospective study of 1129 UFE patients (and at least one visible ovarian artery (OA); 184 (17.2%)) that aortography identified collateral OA supply to more than 10% of the uterus in only 0.8% cases.^30^ Since the selective ovarian arteriography detected only 5.8% of cases as having collateral OA supply, aortography rarely assists in identifying substantial residual OA supply to the uterus and has limited utility in routine practice of UAE.^30^ Consequentially, limiting the use of aortography has the capacity to reduce dose.

### Radiologist and radiographer expertise

The interventional radiologist and the radiographer have the joint capacity to control the angiography x‐ray unit and to minimise radiation exposure to the patient during a UAE procedure. The radiologist has the training to use angiographic devices such as catheters, wires and embolisation mechanisms and these skills have an overall impact on procedure time and total radiation exposure. Xu *et  al* 31 found that there was a learning effect associated with radiation exposure during cerebral angiography procedures performed by trainee radiologists, where this learning effect was significant with increased radiation dose during the earlier procedures. This was possibly attributed to by the insufficient catheter manipulation skills of novice trainees causing increased fluoroscopy time and dose.^31^ Moreover, Das *et  al* 32 conducted a retrospective analysis of UAE procedures and the respective radiation dose and fluoroscopy times that were performed by trainee interventional radiologists at an interventional radiology training unit. The parameters were categorised into three groups: Group 1, first five UAE cases; Group 2, >5 UAE cases; and Group 3, last five UAE cases. It was found that the mean fluoroscopy time was 18.4, 17.3 and 16.3 min for Groups 1, 2 and 3 respectively. The DAP was 4955 Gy cm^2^ for Group 1, 4583 Gy cm^2^ for Group 2 and 4943 Gy cm^2^ for Group 3. The outcomes of the research did not produce any statistically significant results between the groups (*P *>* *0.05) with fluoroscopy time or radiation dose.^32^ Compared to the study by Xu *et  al* 31, there was no learning curve identified for the trainee interventional radiologists and shows that for a standardised procedure such as UAE, the trainees have the potential to match the skills of primary operators in terms of interventional technique and dose reduction.

### Radiologist and operator technique

Bratby *et  al* 33 compared the effectiveness of unilateral and bilateral transfemoral punctures for UAE and their impact on fluoroscopy time, patient dose and examination complexity. The mean fluoroscopy time in the 12 patients with elective bilateral punctures was 12.8 min, compared with 16.6 min for the other twelve patients with unilateral puncture. There was no statistically significant difference noted in overall procedure time (*P *=* *0.68) between the two transfemoral access methods.^33^ A publication by Maleux *et  al* 34 in 2014 focused on radiologists’ preferred technique of 3D roadmapping for uterine artery visualisation and access. This study aimed to evaluate the validity of 3D roadmapping compared to conventional 2D roadmapping for UFE cases and assess the techniques effect on radiation dose and total procedure time. No previous studies had investigated the clinical efficacy and radiation dose of rotational angiography 3D roadmap with UFE procedures. Their results showed no significant difference in estimated ovarian dose between the patients randomised into the 3D and 2D intervention group (0.12 vs. 0.15 Gy); *P *=* *0.07) and that the procedure time was shorter when conventional 2D roadmapping was employed (*P *=* *0.01). The total DAP was less for 3D roadmapping versus 2D roadmapping (437 vs. 652 Gy cm^2^; *P *=* *0.07).^34^ The selective 3D rotational angiography of the internal iliac arteries and terminal branches is an effective imaging tool during UFE that does not attribute more dose than 2D roadmapping, however, its use is primarily dependent on radiographer and interventional radiologist preference.

### Modern angiography x‐ray units

Modern and emerging interventional angiography suites have changed from conventional image intensifiers and fluoroscopy units, to c‐arm angiographic systems with flat–panel detectors and integrated automatic exposure control (AEC). Performing a UAE procedure using flat–panel technology yields a wider dynamic range, improved modulation transfer function and decreased image lag compared to image intensifier angiography.^35^ These improvements have the potential to yield enhanced image quality and result in lower radiation exposure to the UAE patient. Sapoval *et  al* 36 conducted a study to assess the ability of low–dose and low–frame rate fluoroscopy and angiography using a flat–panel detector angiographic suite (Axiom Artis, Siemens Medical Systems, Germany) to reduce the radiation exposure to patients undergoing UFE. One UFE group were performed with standard fluoroscopy (15 pulses/sec) and angiography (3 frames/sec) and a second UFE group were imaged using low–dose/low–frame rate fluoroscopy (7.5 pulses/sec for catheterisation and 3 pulses/sec for embolisation) and angiography (1 frame/sec). For the following parameters measured, the authors found notable reductions in dose due to flat–panel technology; mean peak skin dose (2.4–0.4 Gy (*P *=* *0.001)), DAP (431.13–95.15 Gy cm^2^ (*P *=* *0.003)), ovarian dose (0.378–0.083 Gy), uterus dose (0.388–0.085 Gy) and effective dose (112 to 24 mSv (*P *=* *0.003)).^36^ These findings demonstrate the positive effects of technological advancements on reducing radiation exposure and further promoting the ALARA principle.

In 2013, Firouznia *et  al* 37 further explored the ovarian radiation doses in flat–panel and conventional angiography during UAE by performing a randomised trial. Thirty women were randomised into two UAE treatment groups using either a conventional DSA unit (Advantx, GE Medical Systems, Illinois, USA) or a flat–panel detector system (Innova 4100, GE Medical Systems, Illinois, USA). The ovarian doses were derived from in vitro phantom study calculations. Their measurements showed that the mean right side ovarian dose was 0.14 ± 0.09 Gy for the conventional DSA group and 0.026 ± 0.02 Gy for the flat–panel detector group (*P *=* *0.0001) and the mean left ovarian dose was 0.10 ± 0.08 Gy for conventional DSA and 0.02 ± 0.02 Gy for the flat–panel group (*P *=* *0.002).^34^ These results suggest that the use of flat–panel angiography systems not only improves the diagnostic image quality, but also reduces the overall radiation exposure to the patient due to the improvements in the detective quantum efficiency (DQE).^38^, ^39^ Enhanced image quality allows the interventional radiologist to more easily visualise and perform their interventional techniques, which effectively reduces the total fluoroscopy time and radiation dose. This study was able to depict the benefits of flat–panel technology on ovarian dose but did not document the effect on cumulative DAP and air kerma (AK).

Due to the advent and presence of flat‐panel x‐ray units in the angiography suites, several studies have been published to date which compares the use of this technology to conventional angiography units during UAE. Most of the literature revolves around radiation dose and the common method of measuring and recording the AK, DAP and fluoroscopy time.^9^, ^10^, ^28^, ^30^, ^35^, ^36^ Such methods and analysis improve upon the limitations of findings by Firouznia *et  al* 37. Kohlbrenner *et  al* 40 and Sommer *et  al* 41 independently investigated the radiation dose associated with using an optimised processing and acquisition platform. The former study retrospectively analysed the radiation dose data for 21 patients who had a UFE procedure using a conventional angiography unit and 49 patients performed on a modern angiography system.^40^ Kohlbrenner *et  al* 40 found that the mean CKAP (cumulative kerma‐area‐product) decreased by a considerable 60% from 438.5 to 175.2 Gy cm^2^ (*P *<* *0.0001) and the mean CAK (cumulative air kerma) decreased by 45% from 2.03 to 1.21 Gy (*P *=* *0.001). The latter study by Sommer *et  al* 41 had a larger cohort of 286 patients who were divided into two groups undergoing UFE with a flat–panel (Group 1) or conventional (Group 2) angiography unit. The results showed a DAP reduction in Group 1 (11.59 Gy cm^2^; *P *<* *0.001) compared to Group 2 (31.24 Gy cm^2^).^41^ Both studies produced notable statistically significant outcomes with minimising radiation exposure to the UFE patients and demonstrates that flat–panel angiography units are more superior to conventional angiography units. Another study by Mondshine *et  al* 35 also found that the technology was associated with a decreased cumulative dose (0.78 Gy) and skin DAPs (146.35 Gy cm^2^) on their flat–panel angiography system (Axiom Artis, Siemens Medical Systems, Germany). Recent literature by Schernthaner *et  al* 42, published in 2018 supports previous findings that the flat–panel angiography units produce significantly improved image quality and reduced radiation exposure. These benefits further justify the clinical viability of UAE as a non‐invasive procedure and offset any potential, but rare, risks that may be involved with this technique.^43^

### Future considerations

From the reviewed literature, measurement of UAE radiation exposures has been accounted for by either direct measurement using TLDs or by measuring the DAP, AK and cumulative effective dose directly from the angiography x‐ray unit.^24^ As shown in Figures 2 and 3, reductions in radiation dose exposures are evident when an intervention such as using dose optimisation techniques or a new angiography x‐ray unit was used. A dose reduction trend overtime may not be seen due to the different interventions used, mixed sample sizes, operator and machine variability, case complexity and the changes in interventional treatment when better quality diagnostic imaging is available with upgraded technology. The future direction of research on radiation exposure and UAE involves the continuation of the studies in comparing dose differentials between older and new angiography x‐ray units that are equipped with real–time image processing techniques and dose reduction algorithms.^44^ The current trend in interventional radiology is with the Transradial Approach (TRA), where Resnick *et  al* 45 demonstrated that TRA‐UAE is safe and feasible with patent radial artery at 1‐month follow‐up in all patients. Research into the TRA could potentially reduce procedure time and consequently, fluoroscopy time and radiation exposure to the patient. Other studies into different types of angiography catheters (RUC, C1, C2 and microcatheters) and pre‐UAE procedure weight loss would be beneficial.

## Conclusion

In summary, UAE is a viable procedure for the treatment of symptomatic fibroids and/or adenomyosis utilising minimally invasive angiographic techniques and image guidance with minimal radiation dose. The reviewed literature does not identify any immediate stochastic or deterministic effects of radiation exposure to the patient, however a longitudinal study on any long‐term radiation–induced consequences such as skin injury or cancer risk, post‐UAE would be recommended. The total radiation exposure of UAE patients are affected independently by multiple patient, operator expertise and technique, angiographic imaging and x‐ray unit variables. Application of the ALARA principle during UAE procedures allows for safe radiation practice while achieving optimal clinical results for potentially fertile patients. The literature has shown that reducing frame rates, collimation, PA projections, minimal DSA acquisitions, intermittent fluoroscopy and use of refined FPD technology and dose optimisation software are methods for reducing the radiation dose. Future research involves finding a correlation in DAP measurements with TLD values to account for absorbed dose and continual analysis of dose contributors in current UAE practice to minimise the total radiation exposure on UAE patients within their reproductive age.

## Conflict of Interest

The authors declare no conflict of interest.
